# Sudomotor dysfunction reflects early atherosclerosis risk in adults with type 1 diabetes

**DOI:** 10.1038/s41598-026-36292-w

**Published:** 2026-01-16

**Authors:** Dariusz Naskręt, Agnieszka Gandecka-Pempera, Michał Kulecki, Aleksandra Araszkiewicz, Dorota Zozulińska-Ziółkiewicz

**Affiliations:** 1 Department of Internal Medicine and Diabetology, Poznan University of Medical Sciences, Poznan, Poland; 2 Doctoral School, Poznan University of Medical Sciences, Poznan, Poland

**Keywords:** Diseases, Endocrinology, Medical research

## Abstract

Adults with type 1 diabetes (T1D) are at increased risk of premature atherosclerosis. Sudomotor dysfunction (SMD), an early manifestation of diabetic neuropathy, may contribute to vascular injury. This cross-sectional study assessed the relationship between sudomotor function (SMF), carotid intima-media thickness (cIMT), and vascular age (VA) in T1D. The study included 299 adults with T1D (137 men), aged 34 (IQR: 25–44) years, disease duration 16 (IQR: 11–25) years, and HbA1c of 7.7 (IQR: 7.0–8.7)%. Sudomotor function was measured with the SUDOSCAN device; abnormal function was defined as Feet ESC < 70 µS (SMD). cIMT was assessed with carotid ultrasound, and VA was derived from cIMT values. Participants with SMD had thicker cIMT [0.56 (IQR: 0.5–0.67) vs 0.54 (0.48–0.52), p = 0.04] and higher VA [48 (36–70) vs 42 (32–58), p = 0.04]. We found a negative correlation between Feet ESC and cIMT (Rs = − 0.22, p < 0.001). In a multiple linear regression model adjusted for sex, HbA1c, BMI, and creatinine, reduced Feet ESC remained significantly associated with VA (β = 0.13, p = 0.03), R^2^ = 0.065. SMD is associated with increased cIMT and VA in adults with T1D. SMF assessment by SUDOSCAN may represent a rapid, non-invasive tool to identify individuals at higher cardiovascular risk.

## Background

Type 1 Diabetes (T1D) is a chronic disease caused by absolute insulin deficiency due to autoimmune destruction of pancreatic β-cells. Cardiovascular diseases (CVD) are the leading cause of death, accounting for approximately 35% of deaths and nearly 50% of years of life lost in T1D^1,2^. In recent decades, significant improvements in diabetes management have caused an extension of life expectancy in individuals with T1D^3–5^. Nevertheless, the mortality risk in this population remains two to five-fold higher compared with individuals without T1D^6^. The mechanisms underlying accelerated atherosclerosis in T1D are still under investigation. Even individuals with well-controlled glycemia and without classical cardiovascular risk factors have an elevated risk of developing CVD, suggesting additional diabetes-specific pathophysiological pathways. These include chronic inflammation, oxidative stress, and endothelial and microvascular dysfunction^7^. Early identification of subclinical vascular damage is therefore critical for CVD prevention. Carotid intima-media thickness (cIMT), measured by high-resolution ultrasonography, is a non-invasive, well-established marker of subclinical atherosclerosis and a strong predictor of incident cardiovascular events in both the general population and in T1D^8–11^. Moreover, estimating vascular age (VA) based on cIMT measurements provides additional prognostic value for identifying individuals at high risk of CVD^12^.

Microvascular complications are also considered risk factors for CVD^13–15^. The Diabetes Control and Complications Trial/Epidemiology of Diabetes Interventions and Complications Study (DCCT/EDIC) demonstrated that the presence and progression of micro- or macroalbuminuria independently predicted first cardiovascular events in T1D^16^. Peripheral and autonomic neuropathies contribute to cardiovascular dysregulation by disrupting heart rate control, blood pressure variability, and coronary perfusion^17,18^. Sudomotor dysfunction (SMD), reflecting early small-fiber neuropathy, represents an additional microvascular-related abnormality. SMD is a consequence of impaired function of thin, unmyelinated C-fibers innervating the sweat glands. SUDOSCAN device assesses it non-invasively by measuring electrochemical skin conductance^19–21^. Thin, unmyelinated C-fibers innervate sweat glands. The SUDOSCAN device can assess their function simply, reproducibly, and non-invasively by measuring electrochemical skin conductance. Emerging evidence links SMD to subclinical atherosclerosis in type 2 diabetes and even in non-diabetic individuals^22,23^. However, data on the T1D population remains limited.

### Objective

This study aimed to investigate the relationship between cIMT, VA, and sudomotor function (SMF) in individuals with T1D.

## Participants and methods

### Setting and sample

This cross-sectional study included 299 participants with T1D. Inclusion criteria were: antibody-confirmed T1D, age > 18 years. Exclusion criteria were: pregnancy, active foot ulceration or limb amputation, ketoacidosis, acute infection, implanted electronic devices, mental and neurological disorder, and diagnosed CVD. We confirm that all methods were performed in accordance with the relevant guidelines and regulations, and approved by the Bioethical Committee of the Poznan University of Medical Sciences (approval no. 67/19). Our study conforms to the principles outlined in the Declaration of Helsinki. Written informed consent was obtained from all participants before inclusion in the study.

Each study participant completed a questionnaire that collected information on demographics, T1D history, complications, comorbidities, treatment, and smoking status. All participants underwent a physical examination, including assessment of anthropometric parameters (body weight, height, hip and waist measurement) and blood pressure (BP) measurements following the European Society of Hypertension Guidelines. Hypertension (HT) was diagnosed if the average BP was equal to or above 140/90 mmHg or in individuals with a history of HT^24^. Fasting blood samples were collected to assess HbA_1c_, thyroid-stimulating hormone (TSH), creatinine, C-reactive protein (CRP), and lipid profile.

Skin autofluorescence (SAF) was measured with an AGE Reader (Type 214D00102; DiagnOptics, Groningen, the Netherlands)^25^.

### Carotid intima-media thickness (cIMT) and vascular age (VA) assessment

The carotid imaging was performed by a cardiologist with extensive experience in vascular ultrasonography using a GE Vivid S6 Ultrasound Machine (GE Healthcare, Chicago, Illinois, US) with the 2.4–10 MHz Linear Array Probe (GE Healthcare). The measurement of cIMT of the right common carotid artery (CCA) was taken at least 5 mm from the bulb on a straight 10 mm Section^26^. In arteries, longitudinal images were captured at 16 frames per second for 5 s. cIMT was automatically calculated with the Carotid Analyzer for Research (CAD5) program (Medical Imaging Applications LLC, Coralville, Iowa, US). The results were the average of 100 automated measurements with a high precision of 0.01 mm. Using the Vascular Age Calculator based on cIMT, age, and sex (https://www.quipu.eu/vascular-age-calculator), we calculated VA, which expresses the age of the arteries based on cIMT reference intervals from healthy populations^12^.

### Sudomotor function assessment

Sudomotor function (SMF) was evaluated using the SUDOSCAN device (Impeto Medical Inc., Paris, France), which provides a rapid and non-invasive assessment of electrochemical skin conductance (ESC). The device uses stainless-steel electrodes for hands and feet, and a low-voltage current (< 4 V) is applied during the approximately 2-min measurement. ESC, expressed in microsiemens (µS), reflects the electrochemical reaction between chloride ions in sweat and the electrodes. A Feet ESC value < 70 µS was used to define sudomotor dysfunction based on prior validation studies demonstrating its diagnostic utility for detecting small-fiber neuropathy, particularly the work by Vinik et al. study^27^, in addition to the manufacturer’s recommendation.

### Statistical analysis

We used the STATISTICA 13.0 program for statistical analysis. All data are expressed as medians and interquartile range or percentage of the cohort. Normality of continuous variables was evaluated using the Kolmogorov–Smirnov test with Lilliefors correction, supplemented by visual inspection of histograms. Comparison of two groups divided by Feet ESC and cIMT was performed using the Mann–Whitney test for continuous variables and the Chi-square test for categorical variables. Effect sizes for Mann–Whitney tests were reported using the rank-biserial correlation (r) and Cliff’s delta (δ). The correlation between the conductivity of the skin and cIMT, VA, clinical characteristics, metabolic control, and inflammatory markers was evaluated using Spearman’s correlation coefficient. A multiple linear regression model was used to reveal the association between ESC and VA. Variables for the multivariable linear regression were selected based on the results of univariable analyses. Missing data were assessed for all variables. Most variables had no missing values. WHR had 1 missing value; SBP, DBP, total cholesterol, and CRP had 3 missing values each. Daily insulin dose had 24 missing observations, and SAF measurements were available for 135 participants (164 missing). Because the proportion of missing data was low for clinical and biochemical variables, a complete-case analysis was performed. Results with a probability value < 0.05 were considered statistically significant.

## The results

**Fig. 1:**
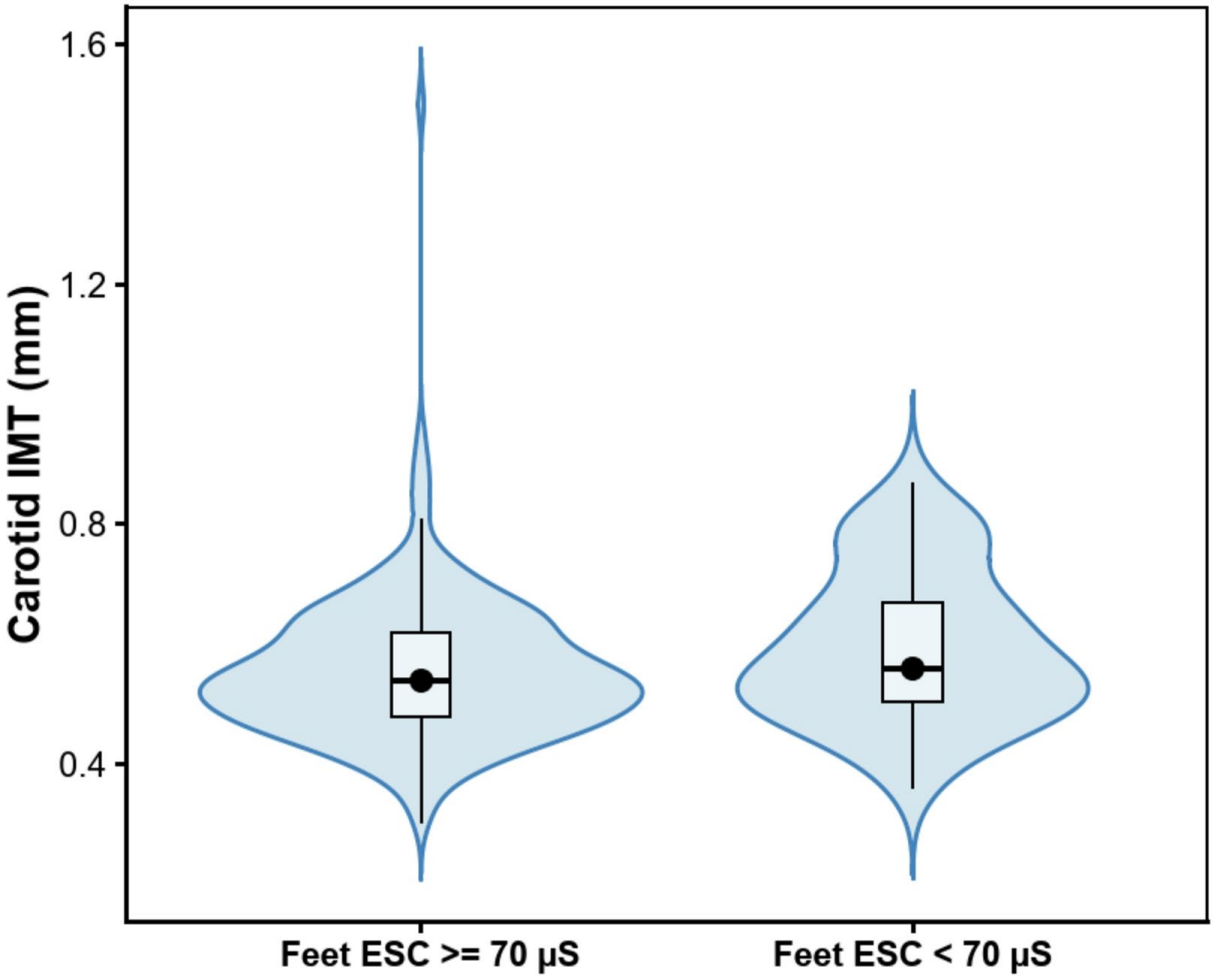
Violin plots showing the distribution of carotid IMT in participants with feet ESC ≥ 70 µS and < 70 µS. Black dots indicate medians. Median differences and 95% confidence intervals were estimated using a nonparametric percentile bootstrap (5000 resamples).

**Fig. 2:**
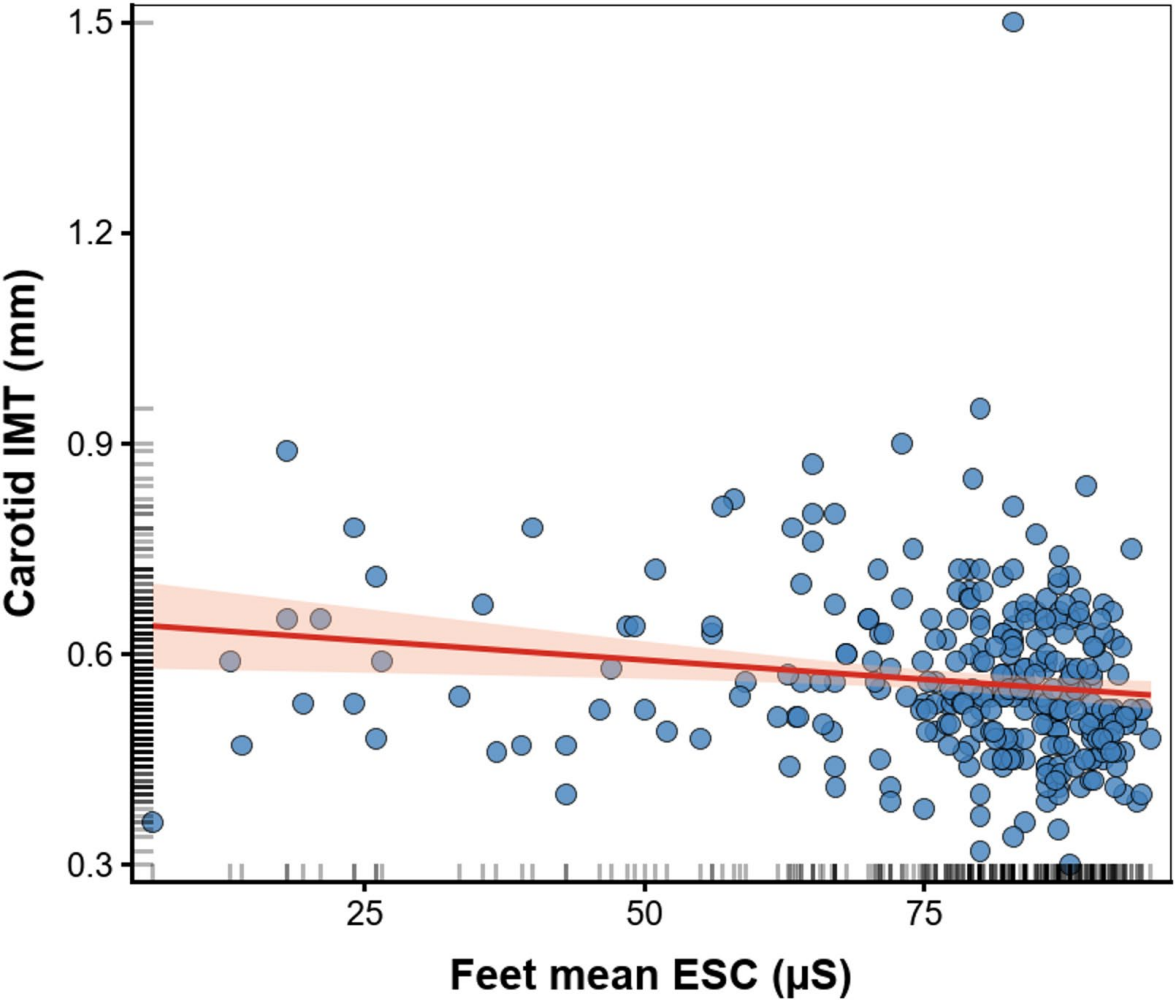
Scatterplot illustrating the relationship between feet electrochemical skin conductance (ESC) and carotid intima–media thickness (IMT). Each point represents an individual participant. The solid line indicates the fitted linear regression, and the shaded area represents the 95% confidence interval of the regression line.

The study included 299 adults with T1D (137 men), age 34 (IQR: 25–44) years, with disease duration of 16 (IQR: 11–25) years and HbA_1c_ of 7.7 (IQR:7.0–8.7) %. In the entire cohort, 41 individuals (14%) had clinically diagnosed Diabetic Peripheral Neuropathy (DPN), 20 (7%) had Cardiac Autonomic Neuropathy (CAN), 92 (31%) had Diabetic Retinopathy, and 24 (8%) had Diabetic Kidney Disease (DKD). A total of 51 participants (17%) had abnormal electrochemical skin conductance, with Feet ESC < 70 µS. The clinical characteristics of the study group are presented in Table 1. Participants with abnormal Feet ESC were older, had a longer diabetes duration, and more often had arterial hypertension. No significant differences were observed between groups in cardiometabolic control parameters, including lipid profile and HbA_1c_. In contrast, individuals with abnormal Feet ESC exhibited higher skin autofluorescence, indicating greater accumulation of advanced glycation end-products (AGEs) (Table 2). Individuals with SMD had thicker cIMT [0.56 (IQR:0.5–0.67) vs 0.54 (IQR:0.48–0.52) mm, p = 0.04] (Fig. 1) and higher VA [48 (36–70) vs 42 (32–58) years, p = 0.04] (Table 2). We found a negative correlation between Feet ESC and cIMT (Rs = − 0.22, p < 0.001) (Fig. 2), and consequently between Feet ESC and VA (Rs = − 0.23, p < 0.001) (Table 3).

**Table 1:** Clinical characteristics of the study group (N = 299).

Characteristic	
Women/Men [n] (%)	162/137 (54/46)
Age [years]	34 (25–44)
Vascular Age [years]	44 (32–60)
Duration of diabetes [years]	16 (11–25)
Smoking [n] (%)	74 (25)
Hypertension [n] (%)	87 (29)
cIMT [mm)	0.54 (0.48–0.63)
Body weight [kg]	75 (63–86)
BMI [kg/m^2^]	24.9 (22.2–27.8)
Waist circumference [cm]	87 (78–96)
Hip circumference [cm]	102 (97–107)
WHR	0.84 (0.79–0.92)
Daily dose of insulin [u/kg b.w./24 h]	0.54 (0.41–0.65)
HbA_1c_ [%] (mmol/mol)	7.7 (7.0–8.7) 61 (53–72)
TCh [mmol/l]	4.8 (4.2–5.5)
TG [mmol/l]	1.0 (0.8–1.3)
LDL [mmol/l]	2.6 (2.0–3.2)
HDL [mmol/l]	1.7 (1.4–2.0)
Creatinine [mg/dl] (µmol/l)	0.83 (0.74–0.92) 73 (65–81)
eGFR [ml/min/1.73 m2]	105.1 (92.6–115,9)
SBP [mmHg]	125 (120–136)
DBP[mmHg]	80 (70–85)
SAF [arbitrary units]	2.2 (1.9–2.8)
hsCRP [mg/l]	1.1 (0.6–2.5)
TSH [uIU/ml]	1.8 (1.2–2.7)
Hands ESC [µS]	71 (62–80)
Feet ESC [µS]	83 (75–88)
Feet ESC < 70 us [n (%)]	51 (17)
Diabetic Peripheral Neuropathy [n (%)]	41 (14)
Cardiac Autonomic Neuropathy [n (%)]	20 (7)
Diabetic Retinopathy [n (%)]	92 (31)
Diabetic Kidney Disease [n (%)]	24 (8)

cIMT, carotid intima-media thickness; BMI, body mass index; WHR, waist hip ratio; TCh, total cholesterol; TG, triglycerides; LDL, low density lipoprotein; HDL, high density lipoprotein; eGFR, estimated glomerular filtration rate; SAF, skin autofluorescence; ESC, electrochemical skin conductance. Values are median (interquartile range) or numbers and percentages.

**Table 2:** Comparison of characteristics according to Feet ESC, data presented as medians with interquartile ranges ( Mann–Whitney test) or numbers and percentages (Chi^2^ test).

Characteristic	Feet ESC > = 70	Feet ESC < 70	P	R	δ
N	248	51			
Women/Men [n] (%)	135/113	27/24	NS		
Age [years]	**32 (24–40)**	**44 (34–54)**	** < 0.001**	− 0.309	− 0.475
Vascular Age [years]	**42 (32–58)**	**48 (36–70)**	**0.04**	− 0.121	− 0.186
Duration of diabetes [years]	**16 (10–23)**	**22 (16–34)**	** < 0.001**	− 0.236	− 0.36
Smoking [n] (%)	58 (23)	16 (31)	NS		
Hypertension [n] (%)	**62(25)**	**25 (49)**	** < 0.001**		
cIMT (mm)	**0.54 (0.48–0.52)**	**0.56 (0.5–0.67)**	**0.04**	− 0.121	− 0.183
Body weight [kg]	75 (63–86)	73 (63–86)	NS	− 0.002	− 0.002
BMI [kg/m^2^]	24.9 (21.9–27.8)	25.4 (22.9–28.4)	NS	− 0.04	− 0.063
WHR	**0.84 (0.79–0.91)**	**0.90 (0.80–0.94)**	**0.01**	− 0.148	− 0.228
Daily dose of insulin [u/kg b.w./24 h]	0.54 (0.41–0.64)	0.55 (0.43–0.69)	NS	− 0.01	− 0.015
HbA_1c_ [%] (mmol/mol)	7.7 (7.0–8.7) 61 (53–72)	7.8 (7.4–8.9) 62 (57–74)	NS	− 0.07	− 0.107
TCh [mmol/l]	4.8 (4.2–5.5)	5.1 (4.5–5.7)	NS	− 0.07	− 0.108
TG [mmol/l]	1.0 (0.8–1.3)	1.0 (0.8–1.6)	NS	− 0.058	− 0.089
LDL [mmol/l]	2.6 (2.0–3.2)	2.8 (2.2–3.4)	NS	− 0.095	− 0.146
HDL [mmol/l]	1.7 (1.4–2.0)	1.7 (1.4–1.9)	NS	− 0.027	− 0.041
Creatinine [mg/dl] [µmol/l]	0.83 (0.75–0.92) 73 (66–81)	0.83 (0.72–0.97) 73 (64–86)	NS	− 0.006	− 0.01
eGFR [ml/min/1.73 m2]	106.1 (93.2–115.6)	100.6 (85.5–117.1)	NS	0.056	− 0.086
SBP [mmHg]	125 (120–135)	130 (120–140)	NS	− 0.103	− 0.158
DBP [mmHg]	80 (70–85)	80 (70–85)	NS	0.015	− 0.023
SAF [arbitrary units]	**2.1 (1.8–2.6)**	**2.7 (2.3–3.1)**	**0.002**	− 0.263	− 0.376
TSH [uIU/ml]	1.8 (1.2–2.6)	1.8 (1.2–2.7)	NS	− 0.015	− 0.023
hsCRP [mg/l]	1.8 (1.0–3.30)	1.3 (0.6–3.8)	NS	− 0.075	− 0.115
Hands ESC [µS]	**73 (66–81)**	**52 (39–62)**	** < 0.001**	0.48	0.738
Feet ESC [µS]	**85 (80–89)**	**56 (36–64)**	** < 0.001**	0.641	− 0.986

Significant values are in bold.

cIMT, carotid intima media thickness; BMI, body mass index; WHR, waist hip ratio; TCh, total cholesterol; TG, triglycerides; LDL, low density lipoprotein; HDL, high density lipoprotein; eGFR, estimated glomerular filtration rate; SAF, skin autofluorescence; ESC, electrochemical skin conductance.

**Table 3:** Correlation between electrochemical skin conductance and clinical characteristics of the study group. Spearman’s coefficient.

Characteristic	Feet ESC			cIMT		
Variables	Rs value	P	p#	Rs value	P	p#
Age [years]	− **0.43**	** < 0.001**	** < 0.02**	**0.57**	** < 0.001**	** < 0.02**
Duration of diabetes [years]	**− 0.42**	** < 0.001**	** < 0.02**	**0.36**	** < 0.001**	** < 0.02**
Vascular age [years]	**− 0.23**	** < 0.001**	** < 0.02**	**0.99**	** < 0.001**	** < 0.02**
BMI [kg/m^2^]	− 0.06	0.26	> 0.999	**0.18**	**0.001**	**0.02**
WHR	**− 0.12**	**0.02**	0.4	**0.23**	** < 0.001**	** < 0.02**
Daily dose of insulin	0.00	0.99	> 0.999	**− 0.22**	** < 0.001**	** < 0.02**
cIMT [mm]	**− 0.22**	** < 0.001**	** < 0.02**			
HbA_1c_ [%] (mmol/mol)	**− 0.12**	**0.04**	0.8	**− 0.14**	**0.02**	0.4
TCh [mmol/l]	**− 0.12**	**0.02**	0.4	0.04	0.5	> 0.999
TG [mmol/l]	**− 0.13**	**0.03**	0.6	− 0.07	0.88	> 0.999
LDL [mmol/l]	− 0.12	0.04	0.8	0.01	0.9	> 0.999
HDL [mmol/l]	0.03	0.52	> 0.99	0.07	0.22	> 0.999
TSH [uIU/ml]	− 0.02	0.68	> 0.999	− 0.07	0.25	> 0.999
CRP [mg/l]	− 0.14	0.02	0.4	− 0.02	0.67	> 0.999
Creatinine [mmol/l]	− 0.07	0.27	> 0.999	**0.15**	**0.02**	0.4
eGFR [ml/min/1.73 m2]	0.12	0.05	> 0.999	**− 0.24**	** < 0.001**	** < 0.02**
SBP [mmHg]	**− 0.19**	**0.001**	**0.02**	**0.22**	** < 0.001**	** < 0.02**
DBP [mmol/l]	− 0.06	0.29	> 0.999	0.04	0.42	> 0.999
SAF [arbitrary units]	**− 0.37**	** < 0.001**	** < 0.02**	**0.32**	** < 0.001**	** < 0.02**
Feet ESC [µS]				**− 0.22**	** < 0.001**	** < 0.02**

Significant values are in bold.

p < 0.05 was considered statistically significant; p# denotes Bonferroni-adjusted p values.

In a multiple linear regression model adjusted for sex, HbA_1c_, BMI, creatinine, VA was independently associated with SMD (β = 0.13, B = 3.94, p = 0.03), R^2^ = 0.065 (Table 4).

**Table 4:** Regression coefficients of multiple linear regression with Vascular Age as dependent variable and sex, BMI, creatinine, HbA_1c,_ and Feet ESC < 70 µS as independent variables, R^2^ = 0.065.

	β	B (95% Cl)	P
Sex (male)	0.07	1.64	0.23
BMI	0.11	0.64 (**− **0.05–1.34)	0.07
Creatinine	0.04	3.37 (**− **7.33–14.08)	0.53
HbA_1c_	**−0.14**	**− 2.57 (-4.51–0.63)**	**0.01**
Feet ESC < 70µS	**0.13**	**3.94 (0.41–7.47)**	**0.03**

Significant values are in bold.

## Discussion

In the present study, we analyzed cIMT and VA in the two groups of T1D adults with preserved or reduced ESC. We found that individuals with abnormal Feet ESC had increased cIMT and higher VA. To our knowledge, this is the first study that investigated the relationship between sudomotor function and surrogate markers of cardiovascular risk, as cIMT and VA, in individuals with T1D.

Cardiovascular disease accounts for approximately one-third of all mortality in individuals with T1D. An accurate cardiovascular risk assessment remains essential for tailoring preventive strategies and guiding therapeutic decisions in this population, including managing hyperlipidemia and hypertension and determining optimal glycemic targets. Increased cIMT is widely recognized as a manifestation of early atherosclerosis and serves as a surrogate marker for the condition of other vascular beds^28–30^. Notably, findings from the DCCT demonstrated that cIMT was associated with the subsequent occurrence of coronary artery events^31^. Previous research has also shown that cIMT is significantly increased in individuals with T1D compared with age-matched non-diabetic controls, suggesting an accelerated atherosclerotic process in this population^32^. Moreover, cIMT is increased in individuals with T1D with microvascular complications compared to those without it^33,34^. Using cIMT, VA can be estimated, which may help identify patients with T1D at high cardiovascular risk^35^. Previous research has demonstrated that VA positively correlates with the Steno Type 1 Risk Engine (ST1RE) score recommended by the 2023 European Society of Cardiology Guidelines^36^—a predictive model based on data from 4,306 adults with T1D to estimate the risk of a first fatal or non-fatal cardiovascular event^37^. Importantly, replacing chronological age with VA in the ST1RE calculation increases the estimated cardiovascular risk and reclassifies a substantial proportion of individuals with T1D into higher risk categories^38^.

The development of CVD in T1D is multifactorial. The risk factors include chronic hyperglycemia, hypertension, dyslipidemia, insulin resistance, and microvascular complications—particularly diabetic kidney disease^39^. In our study, both cIMT and consequently VA have shown significant correlations with age, diabetes duration, BMI, body weight, waist-to-hip ratio (WHR), HbA_1C_, skin autofluorescence (SAF), Feet and Hands ESC, eGFR, creatinine, and systolic blood pressure. Interestingly, no significant associations were observed with lipid parameters. Our research revealed a significant association between VA and Feet ESC after adjusting for HbA_1c_, creatinine, sex, and BMI.

Our findings align with previous studies linking SMD to both microvascular and macrovascular complications. The Sudoscan device is a validated tool for the assessment of small-fiber neuropathy^19^, and impaired sudomotor function has also been shown to correlate with autonomic neuropathy^40^. In a study involving 309 patients with type 2 diabetes (T2D), the utility of the Sudoscan device for assessing microvascular complications was demonstrated^41^. Similarly, in individuals with T2D, SMD is associated with diabetic kidney disease^42^. Evaluation of SMF has also been reported to distinguish patients with diabetic retinopathy from those without microvascular complications^43^. In a previous study, we also reported a correlation between SMD and microvascular complications in a cohort of 404 adults with long-lasting T1D^21^. Microvascular complications, SMD, and increased cIMT may be linked through multifactorial and multidirectional mechanisms. The autonomic nervous system and endothelium-dependent relaxation mediated via the nitric oxide pathway regulate vascular wall tension and blood flow. Small-fiber neuropathy impairs axon-reflex–mediated vasodilation, leading to microcirculatory dysfunction. Peripheral nerve degeneration may result from impaired nutrient delivery due to microangiopathy. Impaired perfusion in small vessels induces local neuronal hypoxia, initiating neurodegenerative processes^44,45^. Hypoxia also promotes oxidative stress and endothelial dysfunction, both of which contribute to the development of atherosclerosis. In the DCCT/EDIC study, individuals with cardiovascular autonomic neuropathy experienced a higher long-term risk of CVD events^17^. Mala et al. also reported that the presence of cardiac autonomic neuropathy (CAN) is associated with increased cIMT in individuals with T1D. The authors suggested that CAN contributes to a proinflammatory state and nocturnal blood pressure non-dipping^46^. Elevated blood pressure during sleep may, in turn, promote vascular injury and accelerate the atherosclerotic process^47^. Chronic metabolic imbalance is a common underlying factor for both micro- and macroangiopathic complications. In our study, the significant correlations with Feet ESC and cIMT were observed for skin autofluorescence—a marker of advanced glycation end-products (AGEs)—rather than for glycated hemoglobin (HbA_1c_), which, in contrast to AGEs, reflects short-term glycemic control. A similar correlation between cIMT and AGEs has been reported by Araszkiewicz et al^48^, further supporting the relevance of cumulative glycation in the development of vascular complications in type 1 diabetes.

There is still limited evidence regarding the relationship between SMF and macroangiopathic complications. Recently, a large study including 1,788 individuals with T2D demonstrated that patients with a mean cIMT ≥ 1.0 mm and/or the presence of carotid plaque had significantly lower Feet ESC values compared with those with cIMT < 1.0 mm. In a multivariate regression model, SMD was independently associated with an increased risk of having cIMT ≥ 1.0 mm^22^. However, these findings from a T2D population cannot be directly extrapolated to individuals with T1D, who are generally younger, leaner, and less frequently affected by dyslipidemia or HT. Similarly, in an extensive study of a general middle-aged and elderly Chinese population (n = 5,076), SMF measured with the EZSCAN device was found to be inversely related to cIMT. Participants were stratified into three subgroups according to EZSCAN value (calculated from ESC and demographic characteristics), and SMD was associated with increased cIMT^23^. Moreover, in a study including 36 individuals with T2D and 20 healthy volunteers, ESC values measured with Sudoscan were positively correlated with the ankle–brachial index (ABI), suggesting that reduced sudomotor function may be linked to the presence of peripheral arterial disease^49^.

A major strength of our study is the relatively large, homogeneous, and well-characterized cohort of individuals with T1D, without prior CVD. Several limitations should nonetheless be acknowledged. We did not perform an a priori sample size calculation, which is a limitation of this study. However, our final sample of 299 participants exceeds the commonly cited rule of thumb for multiple regression (n ≥ 50 + 8 k) proposed by Green (1991), indicating that the sample was sufficient. The cross-sectional design does not allow for causal inference, and a coincidental association between the studied variables cannot be excluded. Longitudinal studies are required to determine whether a decline in ESC precedes and contributes to an increase in cIMT and VA. Moreover, only indirect markers were assessed—although ESC is a sensitive measure of small fiber neuropathy, confirmation by skin biopsy or neurophysiological testing was not performed.

Another limitation relates to using cIMT as a marker of subclinical atherosclerosis. Although cIMT measurement has long been considered a surrogate endpoint for cardiovascular risk, its clinical utility remains debated. According to current cardiology guidelines, routine cIMT assessment is not recommended for cardiovascular risk stratification, as there is insufficient evidence that it provides additional predictive value beyond established risk factors, either in the general population or in individuals with type 2 or type 1 diabetes mellitus^36^. In addition, the lack of standardization across various definitions of cIMT limits comparability and clinical utility. In our study, all carotid ultrasound examinations, including cIMT assessment, were performed on the right common carotid artery by a single experienced cardiologist. Although the literature continues to debate which carotid artery and which segment should be used to evaluate atherosclerosis progression and predict cardiovascular outcomes—with many studies applying different protocols—several meta-analyses have highlighted these inconsistencies^50^. Our cohort consisted of adults with T1DM, with a mean age of 34 years, an average disease duration of 16 years, and no diagnosed cardiovascular disease. Notably, previous studies have demonstrated minimal differences in cIMT values between the left and right carotid arteries in young adults without atherosclerotic risk factors^51,52^. We selected the common carotid artery (CCA) rather than the internal carotid artery for our measurements, as earlier research indicates that cIMT assessed in the CCA has greater prognostic value in individuals with T1DM^31^.

Furthermore, our regression model explained only a small proportion of the variability in VA reflecting the complex and multifactorial determinants of vascular ageing. As our aim was to explore independent associations rather than develop a predictive model, the significant associations observed should be interpreted within this context. Age, diabetes duration and hypertension correlate strongly with ESC and cIMT. When either age, hypertension or diabetes duration was added to the regression model, the association between reduced feet ESC and vascular age was no longer significant, indicating shared variance and limiting the ability to establish ESC as an independent predictor. This multicollinearity is a limitation of our analysis. SMD appears to act as a marker that may reflect long-term metabolic dysregulation and microvascular injury, which are also captured by disease duration and vascular age. ESC should not be interpreted as an independent predictor of vascular age, but rather as a non-invasive indicator ssociated with a higher likelihood of neurovascular complication.

Some between-group differences, including cIMT and vascular age, showed borderline statistical significance (p = 0.04). Although such findings should be interpreted cautiously, their consistency across complementary analyses strengthens their credibility.

In conclusion, our research indicates that impaired sudomotor function is associated with markers of subclinical atherosclerosis (cIMT and VA). SMD may reflect cumulative metabolic and microvascular burden rather than acting as an independent determinant of vascular ageing. In daily clinical practice, detecting SMD in individuals with T1D, using a rapid and non-invasive test performed with Sudoscan device, should prompt further screening for macrovascular complications, including cIMT and VA assessment.

## Data Availability

The datasets generated and analyzed during the current study are available from the corresponding author on reasonable request.
